# National Trends and Outcomes of Metabolic and Bariatric Surgery in Adolescents: Insights from the Israeli National Bariatric Surgery Registry

**DOI:** 10.1007/s11695-026-08820-0

**Published:** 2026-06-26

**Authors:** Noa Gosher, Inbal Globus, Alina Rosenberg, Andrei Keidar, Shai Meron Eldar, Adam Abu-Abeid

**Affiliations:** 1 Division of Surgery, Tel Aviv Sourasky Medical Center, Tel Aviv, Israel; 2 Gray’s Faculty of Health and Medical Sciences, Tel Aviv University, Tel Aviv, Israel; 3 Israel Center for Disease Control, Ministry of Health, Tel Hashomer, Ramat Gan, Israel

**Keywords:** Adolescent bariatric surgery, Sleeve gastrectomy, One-anastomosis gastric bypass, National registry, Pediatric obesity, Follow-up attrition

## Abstract

**Background:**

Severe obesity among adolescents is an increasing global health concern associated with significant metabolic and psychosocial morbidity. Metabolic and bariatric surgery (MBS) has emerged as the most effective treatment for durable weight-loss in selected adolescents; however, national data on trends and outcomes remain limited. We aim to evaluate national trends, characteristics, and outcomes of adolescent MBS in Israel using the Israel National Bariatric Surgery Registry (INBSR).

**Methods:**

We conducted a nationwide retrospective analysis of adolescents (< 18 years-old) who underwent MBS between 2017 and 2024. Data were extracted from the INBSR, a mandatory registry capturing procedures across all centers. Baseline demographics, obesity-related diseases, procedures, and weight-loss outcomes were analyzed.

**Results:**

A total of 460 adolescents underwent primary MBS. Mean age was 16.9 ± 0.8 years and mean body mass index was 46.4 ± 6.8 kg/m², with a female predominance (58.7%). Sleeve gastrectomy (SG) was most common (87.6%). Comorbidity burden was high, including fatty liver disease (60–73.8%), obstructive sleep apnea (~ 51.3%), and type 2 diabetes (~ 20%). SG achieved mean excess weight loss (%EWL) of 56.3% at 6 months, 70.7% at 2 years, and 68.5% at 4 years. Other procedures demonstrated greater weight-loss (~ 80% EWL at 3 years). Follow-up declined, with dietitian visits decreasing from 47.6% at 1 year to 14.5% at 4 years. Sixteen patients required revisional surgery mainly due to recureent weight gain.

**Conclusions:**

Adolescent MBS in Israel results in substantial and durable weight-loss. However, high comorbidity burden and poor long-term follow-up highlight the need for improved multidisciplinary care and retention strategies.

## Key Points

SG is the most common MBS in adolescents in Israel.

Adolescents present to MBS with a relatively high BMI and presence of obesity related diseases.

MBS achieved durable weight loss, reaching 70.7%-80% EWL.

A critical decline in long-term nutritional follow-up was observed.

## Introduction

Severe obesity among adolescents has emerged as a major global health concern, with prevalence increasing steadily over the past two decades. Adolescents with severe obesity face a high burden of metabolic, mechanical, and psychosocial morbidity, including type 2 diabetes (T2D), obstructive sleep apnea (OSA), metabolic associated fatty liver disease (MAFLD), hypertension, and significant impairment in quality of life. Importantly, obesity during adolescence frequently persists into adulthood and is associated with early cardiovascular morbidity and reduced life expectancy [1, 2].

Lifestyle modification and pharmacologic interventions remain the first-line treatment strategies; however, these approaches can be less effective in achieving sustained weight reduction in adolescents with severe obesity [3]. Metabolic and bariatric surgery (MBS) has therefore emerged as the most effective therapeutic option for durable weight loss and obesity related disease resolution in carefully selected adolescent patients [4]. Over the past decade, accumulating evidence has demonstrated that bariatric surgery in adolescents results in substantial and sustained weight loss, along with significant improvements in obesity-related diseases such as T2D, hypertension, and dyslipidemia [5].

Furthermore, current guidelines from the American Society for Metabolic and Bariatric Surgery (ASMBS) and the International Federation for the Surgery and Other Therapies for Obesity (IFSO) recognize MBS as a safe and effective treatment for adolescents with severe obesity when performed within a multidisciplinary framework [6, 7].

Despite growing acceptance of MBS in adolescents, important questions remain regarding procedure selection, long-term durability of weight loss, psychosocial outcomes, and the transition of care from pediatric to adult services. In addition, national patterns of adolescent MBS vary considerably between countries due to differences in healthcare systems, regulatory policies, and referral practices. Population-based registry studies are therefore essential to better understand procedural trends, indications, and long-term outcomes in this population.

Israel maintains a unique nationwide bariatric surgery registry that captures all procedures performed across both public and private medical centers [8]. This registry provides a comprehensive opportunity to evaluate national trends in adolescent MBS.

The aim of the present study was to analyze national trends in MBS among adolescents in Israel using data from the Israel National Bariatric Surgery Registry (INBSR).

## Methods

### Study Population and Data Source

We conducted a nationwide retrospective cohort study utilizing data from the Israel National Bariatric Surgery Registry (INBSR). This study includes all adolescent patients (< 18 years old) who underwent MBS in Israel between October 1, 2017, and December 31, 2024. For clarity of trend analysis and data visualization, data from the final quarter of 2017 were aggregated into the 2018 study period. Data were extracted from the INBSR, a mandatory nationwide database established in June 2013 under the auspices of the Israel Ministry of Health, the Israel Surgical Society, and the Israel Endocrinology Society.

To maintain bariatric surgery privileges and eligibility for reimbursement, all 33 medical centers in Israel (23 public and 10 private) are required to submit monthly data. Data are collected via a standardized electronic questionnaire and undergo routine quality control and validation for accuracy by INBSR personnel. Patient anonymity is strictly preserved at the registry level.

### Patient Eligibility and Clinical Indications

In accordance with the formal Israeli Ministry of Health (MOH) regulations [9], surgical eligibility for adolescents in this cohort was strictly governed by national regulatory criteria. Specifically, eligibility was defined using absolute BMI thresholds and the presence of documented obesity-related diseases (as detailed below), without utilization of age- or sex-specific BMI percentiles.

Candidates were required to meet specific physiological, anthropometric, and behavioral criteria:

1. Physiological and Chronological Maturity: Candidates were required to be at least 13 years of age, with documented skeletal maturity (bone age > 15 years for males and > 13 years for females, indicating > 95% of growth completion). For patients under the age of 16, a specialized MOH-appointed exceptional cases committee was required to authorize the procedure, ensuring the immediate health risks outweighed the risks of early intervention.
2. BMI Thresholds and obesity related diseases: Eligibility was stratified into two primary BMI-based categories according to the Israeli MOH guidelines:
  BMI ≥ 40 kg/m² in the presence of *Mild* obesity related diseases including- Orthopedic impairments, hypertension, gastroesophageal reflux disease (GERD), dyslipidemia, urinary incontinence, intertriginous skin fold infections, and polycystic ovary syndrome (PCOS).
  BMI ≥ 35 kg/m² in the presence of *severe* obesity-related diseases, including- T2D, Severe OSA, or Idiopathic Intracranial Hypertension.

### Preoperative Evaluation and Multidisciplinary Approval

The selection process involved a rigorous multidisciplinary evaluation. The preoperative protocol mandated consultations with a pediatric endocrinologist and a child psychiatrist. Candidates for revisional surgery required additional clinical clearance from a bariatric surgeon. Following initial medical suitability, all patients participated in a minimum 6-month structured weight-loss program within a specialized bariatric center. Eligibility was contingent upon demonstrated adherence to medical recommendations (e.g., vitamin supplementation, CPAP usage) and a formal assessment of the candidate’s (and family’s) capacity for lifelong behavioral modification and informed consent.

### Follow-up and Outcome Measures

Post-operative outcomes were assessed through a multi-dimensional longitudinal framework. Postoperative outcomes are captured in the national registry through routine follow-up data reported by patients’ community family physicians and dietitians. Weight loss efficacy was evaluated using the percentage of Excess Weight Loss (%EWL), assessed longitudinally over a 4-year period. Post-operative follow-up included standardized nutritional monitoring by a dietitian at 6 months, 1 year, 2 years, 3 years, and 4 years. Additionally, a sub-analysis was conducted for patients who underwent conversion or revisional surgery as adults. Long-term mortality data were obtained by cross-referencing INBSR records with the Israel National Population Register.

### Statistical Analysis

Statistical analysis was performed using descriptive statistics to summarize the cohort’s baseline clinical and demographic characteristics. Continuous variables (e.g., age and BMI) were expressed as means +\- standard deviation (SD). Categorical variables (e.g., procedure type, obesity-related diseases prevalence, and revisional indications) were presented as absolute frequencies and percentages.

The %EWL was tracked longitudinally to evaluate weight loss durability. Annual trends in surgical volume and obesity-related diseases prevalence were analyzed from 2018 to 2024. All data processing and visualization were conducted to reflect the transition from pediatric to adult bariatric care.

All data processing, statistical computations, and longitudinal trend analysis were conducted using SAS software, version 9.4.

## Results

### Patient Characteristics and Baseline Demographics

During the study period (2018–2024), a total of 460 primary bariatric procedures in adolescents were documented in the INBSR. The cohort demonstrated a consistent female predominance (58.7%, *n* = 270), though gender distribution showed a trend toward parity by 2024 (50.8% female). At the time of surgery, the mean age remained stable across the study years, ranging from 16.7 to 17.1 years (Table 1).

**Table 1:** Annual Distribution and Baseline Characteristics of the Adolescent Cohort (2018–2024)

Year	*N*	Gender (Female %)	Mean Age (± SD)	Mean BMI (± SD)	BMI Range (Min–Max)
2018*	107	57.9%	17.05 ± 0.94	45.91 ± 4.86	38.0–59.8
2019	79	57.0%	16.83 ± 0.86	45.26 ± 6.56	36.4–70.1
2020	46	67.4%	16.92 ± 0.86	45.96 ± 5.79	35.6–59.9
2021	72	66.7%	17.06 ± 0.74	46.44 ± 6.41	35.1–64.6
2022	41	58.5%	16.75 ± 0.83	48.42 ± 7.10	38.3–66.0
2023	50	54.0%	16.71 ± 0.70	48.30 ± 7.35	37.7–68.2
2024	65	50.8%	16.74 ± 0.70	48.87 ± 7.70	34.9–70.6
Total	460	58.7%	16.90 ± 0.80	46.40 ± 6.80	34.9–70.6

*2018 data includes the final quarter of 2017

*BMI* Body Mass Index

The baseline metabolic profile was characterized by severe obesity, with an overall mean preoperative BMI of 46.4 ± 6.8 kg/m². Notably, a trend toward higher surgical acuity was observed in recent years, with the mean preoperative BMI rising from 45.9 kg/m² in the 2018 period to 48.9 kg/m² in 2024.

### Surgical Volume and Procedural Trends

Annual surgical volume reached a peak in 2018–2019 (*n* = 107 and *n* = 79, respectively), followed by a period of decline with a nadir in 2022 (*n* = 41), before rebounding to 65 procedures in 2024 (Table 2).

**Table 2:** Annual Distribution of Bariatric Procedures (2018–2024)

Year	Total Procedures (*n*)	Sleeve Gastrectomy (SG)	OAGB	RYGB	Other**
2018*	107	89	14	1	3
2019	79	72	4	1	2
2020	46	42	4	0	0
2021	72	61	8	1	2
2022	41	39	2	0	0
2023	50	45	3	1	1
2024	65	55	9	1	0
Total	**460**	**403**	**44**	**5**	**8**

*2018 data includes the final quarter of 2017

** “Other procedures” refers to Single-Anastomosis Duodeno-Ileal Bypass with Sleeve (SADI-S, *n* = 1) and Laparoscopic Adjustable Gastric Banding (LAGB, *n* = 7)

Laparoscopic Sleeve Gastrectomy (SG) remained the predominant procedure throughout the study, though its relative share decreased from 91% in 2019 to 85% in 2024. Conversely, a minor increase in One Anastomosis Gastric Bypass (OAGB) was observed, rising from 4 annual cases in 2019 to 9 cases in 2024. Other procedures, such as Roux-en-Y Gastric Bypass (RYGB), SADI-S, and Gastric Banding, remained rare and showed no discernible change in volume. Endoscopic non-surgical interventions are not captured by the INBSR framework and are therefore excluded from this analysis.

### Baseline Clinical Indications and Obesity-related Diseases

#### Severe Metabolic and Mechanical Obesity-related Diseases

**Fig. 1:**
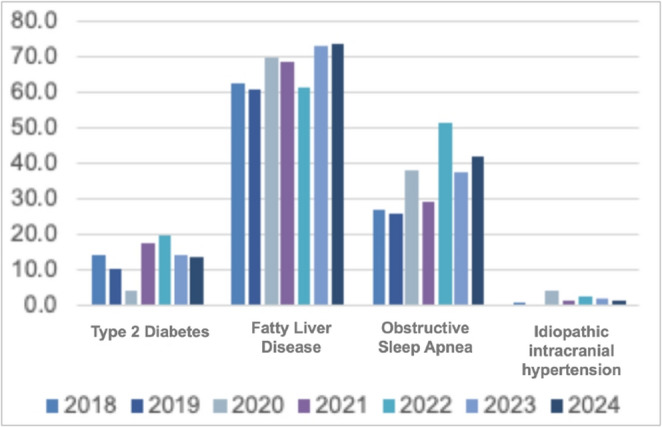
Trends in Severe Obesity-Related diseases as Indications for Surgery / Obstructive Sleep Apnea (OSA) consistently represented as a primary severe indication throughout the study period. Fatty Liver Disease Shows a significant presence in the cohort, often peaking in parallel with the overall rise in adolescent metabolic syndrome. T2D remained a steady but less frequent primary indication compared to mechanical respiratory issues

At the time of clinical presentation, the adolescent cohort exhibited a significant burden of severe obesity-related diseases. MAFLD and OSA were the most prevalent major indications, with MAFLD affecting 60–73.8% and OSA nearly doubling from 27.1% in 2018 to a peak of 51.3% in 2022. While T2D was recorded across all study years, it fluctuated, reaching a high of 20.0% in 2022 before settling at 13.8% in 2024. Notably, idiopathic intracranial hypertension constituted a small but consistent portion of high-severity indications requiring urgent intervention (Fig. 1).

#### Mild Physical Obesity-related Diseases

**Fig. 2:**
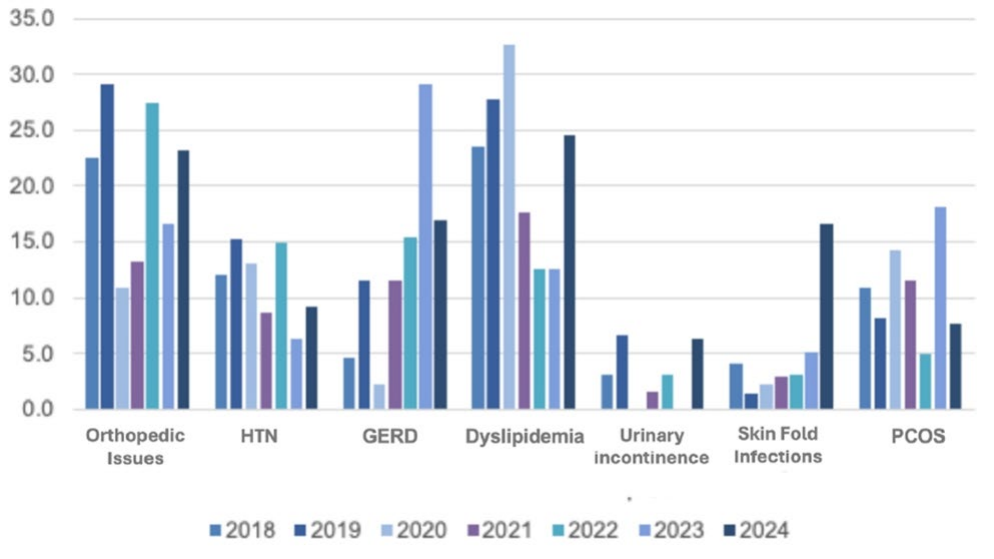
Illustrates the prevalence of mild obesity-related diseases in the adolescent cohort. Orthopedic Issues remained a leading indication, consistently affecting 23.2%-29.1% of candidates annually. Dyslipidemia showed high prevalence, reaching over 32% in 2020, reflecting the significant metabolic burden in this population

As shown in Fig. 2, mild physical indications were highly prevalent at baseline. Orthopedic impairments remained a leading concern, peaking at 29.1% in 2019 and maintaining a consistent presence, with 23.2% of candidates affected in 2024. Dyslipidemia reached its maximum prevalence in 2020, affecting 32.6% of patients, before stabilizing at 24.6% by the end of the study period. GERD exhibited a notable upward trend in the latter half of the study, increasing significantly from 4.7% in 2018 to a peak of 29.2% in 2023. Other persistent obesity-related diseases included hypertension (HTN), which peaked at 15.2% in 2019, and PCOS, which reached a high of 18.2% in 2023. Additionally, urinary incontinence and intertriginous skin fold infections remained steady indicators, with the latter showing a sharp increase to 16.7% in 2024.

### Psychosocial and Neurodevelopmental Profile

Psychosocial screening revealed that ADHD was the most frequent neurodevelopmental condition, exhibiting a steady upward trajectory to a peak of 33.3% in 2024. Eating disorders constituted the second most common baseline condition, showing a marked increase from 15.7% in 2018 to a maximum prevalence of 34.2% in 2023. Notably, there was a substantial surge in affective disorders in recent years; anxiety rose from a baseline of 1.0% in 2018 to 19.2% by 2024, while depression similarly escalated from 5.0% to 17.3% over the same period.

### Weight Loss Outcomes

**Fig. 3:**
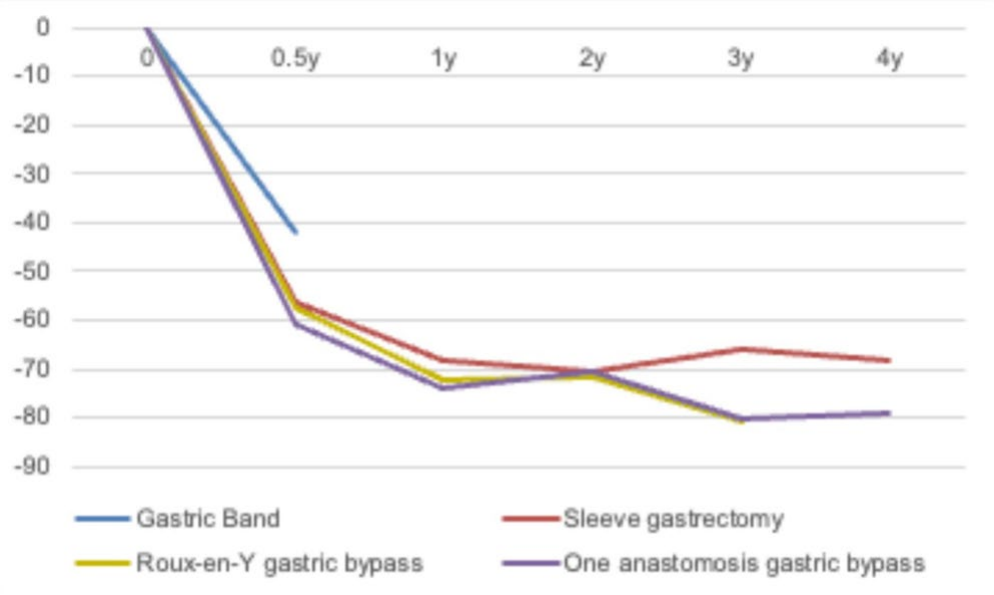
The mean percentage of EWL% over a 4-year follow-up period, stratified by procedure type. Data points are represented at 6 months (0.5y), 1 year (1y), 2 years (2y), 3 years (3y), and 4 years (4y) postoperatively

Longitudinal analysis of %EWL revealed distinct trajectories dictated by procedure type (Fig. 3).

- Malabsorptive and Combined Procedures: OAGB and RYGB demonstrated the highest and most sustained weight loss. OAGB achieved a mean %EWL of 60.9% at 6 months, reaching a plateau of 80.2% at 3 years and maintaining 79.1% by year 4. Similarly, RYGB reached its nadir at 3 years with a mean %EWL of 80.6%.
- Restrictive Procedures: SG, the most frequent procedure (*n* = 403), showed an initial mean %EWL of 56.3% at 6 months, peaking at 70.7% at 2 years. While a modest trend of weight regain was observed at 3 years (66.0%), the cohort stabilized at a mean %EWL of 68.5% by year 4.
- Gastric Banding: Outcomes for gastric banding were notably inferior in the early post-operative phase, with a mean %EWL of only 41.9% at 6 months. Data for this subgroup remained limited in subsequent years due to low procedural volume and high attrition.

Beyond procedural subtypes, the entire cohort demonstrated a robust reduction in total body weight. The mean %TWL was 25.0 ± 8.2% at six months (*n* = 236), reaching a peak of 30.9 ± 9.3% at one year (*n* = 196). This weight loss remained remarkably stable through the mid-to-long term, with mean %TWL values of 31.6 ± 10.9% at two years (*n* = 108) and 29.9 ± 11.7% at four years (*n* = 90).

### Longitudinal Attrition and Nutritional Follow-up

A critical trend of declining post-operative compliance was observed (Table 3). While initial engagement with a dietitian was 47.6% at 6 months and 1 year, retention dropped to 23.8% by year 2. By the fourth year, only 14.5% of the cohort maintained scheduled nutritional follow-up.

**Table 3:** Nutritional Follow-up Attrition Rate (%)

Nutritional monitoring post-op intervals)	2018 (*n* = 107)	2019 (*n* = 79)	2020 (*n* = 46)	2021 (*n* = 72)	2022 (*n* = 41)	2023 (*n* = 50)
6 months (%)	47.6	37.2	47.8	25.0	36.6	39.6
1 year (%)	47.6	36.7	45.7	33.8	34.1	45.8
2 years (%)	23.8	16.9	23.9	20.6	19.5	-
3 years (%)	14.3	6.3	17.8	13.2	-	-
4 years (%)	14.5	12.7	13.0	-	-	-

2018 data includes the last quarter of 2017

### Conversion and Revisional Surgery Analysis

During the follow-up period, 16 patients who underwent SG as adolescents subsequently required conversion/revisional surgery after reaching adulthood (no revisional procedures were recorded during adolescence). The mean age at primary surgery was 16.63 years, and the mean age at revision was 20.49 years (Table 4). The primary driver for conversion \ revision was insufficient weight loss or recurrent weight gain (81.3%, *n* = 13), with a minority revised for surgical complications (18.7%). At the time of conversion, the mean BMI was 42.2 kg/m^2. The most frequent conversion surgery was OAGB (*n* = 8) or RYGB (*n* = 4). Given the small number of revisional cases and limitations of registry-level linkage, we were not able to reliably quantify the association between individual long-term nutritional follow-up and subsequent need for conversion/revisional surgery.

**Table 4:** Profiles of Conversion/Revisional Surgery (Adolescents revised as adults)

Parameter	Primary Surgery (Adolescent)	Revisional Surgery (adult)
Mean Age (Years)	16.63	20.49
Mean BMI (kg/m^2)	48.07	42.2
Primary Procedure	Sleeve Gastrectomy (100%)	-
Revision Reason	-	Recurrent weight gain (*n* = 13); Complications (*n* = 3)
Revision Type	-	OAGB (*n* = 8), RYGB (*n* = 4), SG/band (*n* = 4)

*SG* Sleeve Gastrectomy, *RYGB* Roux en Y Gastric Bypass, *OAGB* One Anastomosis Gastric Bypass

A summary of the literature on national trends in metabolic and bariatric surgery is presented in Table 5.

**Table 5:** Summary of Global National Registries and Large-Scale Cohort Studies Investigating Trends and Outcomes in Adolescent MBS

Study (Authors, Year)	Region	Data Period	Sample Size (*n*)	Participant Ages	Most Frequent Surgery	Surgical Approach	Preoperative obesity-related diseases	Key Clinical Conclusion
Ballinger et al. (2025) [15]	USA (MBSAQIP)	2017–2022	2,229	10–17 years	SG (95% in 2022)	Laparoscopic (85%); Robotic increasing 2x faster	OSA (22%) and T2D (16%); younger teens (13–15) had the highest rates	Robotic cases are surging while complication rates remain low.
Bruze et al. (2024) [38]	Sweden	2007–2017	1,554	< 21 years	RYGB (91.6%)	Standard Laparoscopic	High prevalence of Mental Health issues: Antidepressants (12.4%), ADHD meds (9.6%), and Anxiety (6.9%).	Weight loss does not shift mental health trajectories; alcohol risk increases.
Steinberger et al. (2022) [16]	USA (NIS)	2010–2017	9,014 (Weighted estimate)	12–19 years	SG (3.99 [3.33–4.65] procedures per 100 000)	Minimally Invasive (shift from open- “a frequency too small to report after 2015”)	OSA (25%), GERD (24.6%), and Hypertension (16.2%).	Pediatric surgery is underutilized nationally, particularly among minority groups; preoperative BMI is increasing over time.
Lazzati et al. (2021) [17]	France (PMSI)	2008–2018	8,134 total (1,027 younger; 7,107 older)	< 18, 18–19	AGB (53.5%)/SG (81.7%)	98.1% Laparoscopic	OSA (13.4%), Chronic pulmonary disease (5.6%), and T2D (1.6%).	Despite being safe, there is a progressive decrease in the utilization of surgery for adolescents under 18 in France.
Humayon et al. (2019) [18]	USA (NY, SPARCS)	2005–2014	2,737	12–21 years	SG (73.2% by 2014)	Minimally Invasive (SG)	55.4% of adolescents had at least one obesity-related disease (significantly lower than adults at 81.1%).	Safer outcomes and shorter stays in adolescents compared to adults.
Castellani et al. (2017) [37]	Italy	2000–2010	173	13–18 years	AGB (49.1%)	100% Laparoscopic	43% had obesity-related disease: Steatosis [28], Dyslipidemia [15], Depression [12], and T2D [6].	SG is safe and provides superior weight loss to other restrictive procedures.

This table summarizes key characteristics of major international bariatric registries and large-scale longitudinal studies. *ADHD* attention-deficit/hyperactivity disorder, *AGB* adjustable gastric banding, *GERD* gastroesophageal reflux disease, *HTN* hypertension, *INBSR* Israel National Bariatric Surgery Registry, *SG* laparoscopic sleeve gastrectomy, *MBS* metabolic and bariatric surgery, *MBSAQIP* Metabolic and Bariatric Surgery Accreditation and Quality Improvement Program, *OAGB*-*MGB* one-anastomosis gastric bypass/mini-gastric bypass, *OSA* obstructive sleep apnea, *PMSI* Programme de Medicalisation des Systemes d’Information, *RYGB* Roux-en-Y gastric bypass, *SG* sleeve gastrectomy, *SPARCS* Statewide Planning and Research Cooperative System, *T2D* type 2 diabetes mellitus

## Discussion

In this nationwide analysis of the ISNBR, we evaluated trends, clinical characteristics, and outcomes of MBS in adolescents between 2018 and 2024. Our findings demonstrate that MBS in adolescents in Israel is performed predominantly in patients with severe obesity and a high burden of obesity related diseases. SG is the most common procedure performed (87%) throughout the study period. Surgical outcomes showed meaningful and durable weight loss across all procedures, with hypo absorptive operations demonstrating the greatest magnitude of weight reduction.

Adolescent obesity represents a growing global public health challenge with significant medical, psychosocial, and economic consequences [10]. In 2022, more than 390 million children and adolescents aged 5–19 years were classified as overweight worldwide, including approximately 160 million living with obesity [1]. Severe obesity during adolescence is associated with early onset metabolic and cardiovascular diseases—including T2D, hypertension, dyslipidemia, and MAFLD and frequently persists into adulthood, increasing the lifetime risk of cardiovascular morbidity, malignancy, and premature mortality [11]. In a systematic review of large study cohorts it was reported that approximately 55% of children with obesity remain with obesity in adolescence, nearly 80% of adolescents with obesity continue to have obesity in adulthood, and around 70% remain with obesity beyond the age of 30, highlighting the importance of early intervention [12]. Beyond the physical consequences, adolescents with severe obesity often experience significant psychosocial distress, including depression, anxiety, social stigma, and impaired quality of life [13]. Together, these factors highlight the meaningful long-term burden of untreated severe obesity in adolescents and underscore the importance of effective therapeutic strategies, including MBS in appropriately selected patients.

MBS has emerged as the most effective treatment for adolescents with severe obesity when lifestyle and pharmacologic therapies fail [14]. In our nationwide cohort, SG was by far the most commonly performed procedure, reflecting both international trends [15–18] and a generally a more conservative surgical approach in younger patients. In a retrospective analysis of children and adolescents undergoing sleeve gastrectomy, patients aged ≤ 13 years achieved an excess BMI loss of 54% after 1 year, compared with 44% among the older cohort (*P* = 0.34), with no postoperative complications reported within 30 days in either group [19]. A recent meta-analysis published by Ahmed et al. [20] including 14 studies and nearly 14,000 adolescent patients compared the outcomes of the three commonly performed procedures: SG, RYGB, and adjustable gastric banding (AGB). The analysis demonstrated that both SG and RYGB achieve significantly greater weight loss compared with AGB, while RYGB produced the greatest reduction in weight and BMI overall. However, RYGB was associated with a higher risk of postoperative complications, whereas SG showed a more favorable safety profile. These findings suggest why SG has become the preferred MBS in adolescents despite the potentially greater weight loss observed with hypo absorptive procedures.

One of the most concerning findings of our study is the marked decline in long-term postoperative nutritional and dietitian follow-up among adolescents undergoing MBS. While nearly half of patients remained engaged in nutritional follow-up during the first postoperative year, this rate dropped sharply over time, with only 14.5% maintaining follow-up at four years. In an adult cohort study evaluation predictors for attrition after MBS, Sala et al. [21] reported that out of 397 patients, only 26.2% attended their 2 year follow-up. When assessing the predictors of attending the 2-year follow-up, gastric band and attendance at the 6-months follow-up were found to be significant. Larjani et al. [22] reported in a cohort study that patients older than 25 years demonstrated higher adherence rates compared with younger patients (63.2% vs. 37.5%, *P* = 0.040). This pattern of attrition has significant clinical implications, as consistent long-term monitoring is essential for the early detection and management of nutritional deficiencies, recurrent weight gain, and psychosocial challenges. Although it is intuitive to hypothesize that poor long-term nutritional follow-up might contribute to suboptimal weight loss and subsequent conversion/revisional surgery, our registry did not allow for a robust assessment of this relationship at the individual level, and the small number of revisional cases further limited statistical power.

Several strategies have been proposed to improve long-term follow-up among adolescents undergoing MBS. In the TEEN-LABS cohort, Rode et al. [23] described a structured retention approach and reported high follow-up rates, with in-person visit attendance averaging 86% (95% CI: 85%–87%) and 88% (95% CI: 87%–90%) of patients remaining in follow-up at 10 years. Additional strategies have also been proposed to improve long-term follow-up adherence, including the use of telemedicine, remote monitoring, and, in selected cases, home-based visits. Other approaches such as structured transition programs from pediatric to adult care, digital health platforms, and dedicated multidisciplinary follow-up teams may further enhance retention [23–26]. Collectively, these strategies appear promising and should be considered by bariatric surgeons and multidisciplinary teams to improve long-term adherence and optimize patient outcomes. When interpreting the follow-up data, the relatively high attrition rate should be acknowledged as a potential source of bias. First, the national registry is primarily based on data reported from community health maintenance organizations; therefore, outcomes of patients who continue follow-up in the private sector may not be captured, leading to incomplete data. Second, the unique sociocultural context in Israel, where most adolescents are conscripted into mandatory military service at the age of 18, further contributes to loss to follow-up, as medical care during this period is often delivered outside the civilian healthcare system and may not be consistently reported to the registry. Finally, adolescents as a population are inherently more challenging in terms of long-term adherence to medical follow-up, with lower compliance rates compared to adults, as demonstrated in prior literature. Collectively, these factors may result in underreporting and potential inaccuracies in the database and should be considered when interpreting the findings.

When interpreting the national trends in adolescent MBS, it is essential to acknowledge the unique physiological and utilization patterns that differentiate this group from the adult cohort. First, there is a disproportionately low utilization of metabolic and bariatric surgery (MBS) among younger patients. In the United States, while approximately 250,000 to 280,000 bariatric procedures are performed annually for adults, only 1,600 to 2,000 adolescents undergo MBS each year, representing less than 1% of the eligible pediatric population [15, 16]. This disparity is mirrored in our national findings; we identified only 460 adolescent procedures over seven years, in contrast to the 42,296 adult procedures performed in Israel during a similar period [27]. This ‘treatment gap’ often results in adolescents presenting with higher disease severity by the time they reach intervention; our cohort presented with a mean preoperative BMI of 46.4 ± 6.8 kg/m^2^, a trend consistent with recent US data where mean BMI in surgical cohorts remains above 45 kg/m^2^ [28].

When these patients finally reach the operating table, procedural selection further highlights a distinctly conservative approach compared to adults. While One-Anastomosis Gastric Bypass (OAGB) has rapidly become the leading procedure for adults in Israel—rising from 0.1% in 2014 to 72.6% by 2022 [29–31]—the pediatric trend remains cautious, with our adolescent cohort primarily treated with sleeve gastrectomy (SG) (87.6%). It is likely that this caution is driven by concerns regarding the potential impact of malabsorptive procedures on nutritional status and bone-mass development during critical growth years. Long-term data from the Teen-LABS study (5–11 years post-intervention) demonstrated that young adults who underwent MBS as adolescents had significantly lower dual-energy x-ray absorptiometry (DXA) BMD at the hip (–10.0% for RYGB; − 6.3% for SG) and femoral neck (–9.6% for RYGB; − 5.7% for SG) compared to matched controls (*P* < 0.001) [32]. These longitudinal findings are supported by a recent meta-analysis of 12 studies (681 patients) which found that both SG and RYGB are associated with significantly lower lumbar BMD (–0.96 g/cm², *P* < 0.001) and Z-scores (–1.132, *P* < 0.001) postoperatively [33]. These metabolic and skeletal trade-offs, alongside the problematic attrition rate observed in this population, may explain the relative lack of clinical enthusiasm for transitioning toward more potent interventions like OAGB in the pediatric population, as these procedures are well-recognized for potential complications such as malabsorption and chronic micronutrient deficiencies.

However, emerging data suggest a shift toward more potent interventions may be warranted. Goldenshluger et al. [34] demonstrated that Israeli adolescents experience significant improvements in physical summary scores based on validated quality-of-life (QOL) questionnaires (from 43.4 to 54.1, *P* < 0.001) post-MBS, regardless of comorbidity resolution. Furthermore, Sorek et al. (2023) showed that OAGB achieved superior weight loss at one year compared to SG (86.7% vs. 70.4\% EWL; *P* = 0.007) without increasing major complications [35]. These findings align with a recent international expert consensus where 87.1% of experts agreed that OAGB is a safe primary option for adolescents, provided rigorous nutritional monitoring is maintained [36]. Our study confirms that within the subset of patients who required conversion/revisional surgery after transitioning to adult care, a suboptimal clinical response/recurrent weight gain were the primary driver for conversion surgery in 81.3% of cases. Collectively, these data suggest that moving toward OAGB in high-BMI adolescents may optimize long-term outcomes while maintaining an acceptable safety profile.

As part of our analysis, we conducted a systematic and comprehensive comparison of major international registries and large-scale cohorts, summarized in Table 5. This comparison underscores several key global trends that frame the Israeli experience. Most notably, there is a global convergence toward SG: across diverse healthcare systems—from the US (MBSAQIP) to France (PMSI) —there is a nearly universal shift toward SG as the primary adolescent procedure. Sweden remains a notable outlier with its preference for RYGB. recent global data suggest that the relatively technical simplicity and reduced nutritional risk of SG have established it as the most popular surgery for pediatric populations. Furthermore, despite a relatively safety profile with low complication rates [18], MBS remains underutilized worldwide in adolescents. The table reveals that even in large healthcare systems like the US, only a fraction of eligible adolescents undergo surgery, often presenting only after significant metabolic deterioration [16]. Finally, the decline in long-term follow-up is not unique to Israel; comparative data from Italy and the US [18, 37] confirm that retention remains a global “Achilles’ heel” in adolescent care.

### Strengths and Limitations

This study has several notable strengths. First, this study is based on a large, high-fidelity national registry that mandates reporting for all bariatric procedures nationwide. Second, while Israel is a relatively small country, our study cohort represents a substantial population of adolescents, offering a robust statistical power that is truly representative of the national clinical landscape. Finally, this work provides a comprehensive comparison to date between a national registry and other international cohorts (as detailed in Table 5).

Nevertheless, several limitations should be acknowledged. As a retrospective analysis based on the INBSR, there is an inherent reliance on the accuracy of registry reporting or no reporting, which may lead to the underestimation of minor, late-onset complications. Furthermore, the high attrition rate in long-term follow-up—while reflecting a universal challenge in adolescent care—limits our ability to provide a complete metabolic profile for the entire cohort at the four-year mark. Additionally, the study lacks granular data on long-term psychosocial resolution and quality-of-life metrics beyond clinical weight loss.

## Conclusions

In this nationwide registry analysis, MBS in adolescents was associated with substantial and generally durable weight loss across procedures, but long-term clinical endpoints beyond weight were incompletely captured. The strategic preference for SG reflects a conservative, staged surgical philosophy intended to balance efficacy with concerns about nutritional and skeletal risks in a population with high baseline psychosocial burden and suboptimal long‑term follow‑up. These findings underscore both the potential benefits and the limitations of current practice and highlight the need for structured, multidisciplinary care pathways and improved retention strategies to ensure the lifelong safety and effectiveness of adolescent MBS.

## Data Availability

No datasets were generated or analysed during the current study.
